# RSV burden and prevention in children in LMICs

**DOI:** 10.1016/S2214-109X(24)00289-4

**Published:** 2024-08-28

**Authors:** Adam MacNeil, Meredith McMorrow

**Affiliations:** Coronaviruses and Other Respiratory Viruses Division, National Center for Immunization and Respiratory Diseases, US Centers for Disease Control and Prevention, Atlanta, GA 30333, USA

The RSV GOLD—ICU Network study, published by the RSV GOLD—ICU Network collaborators in this issue of *The Lancet Global Health*,^1^ showed consistent respiratory syncytial virus (RSV) positivity (614 [29·0%; range 23·0–38·2] of 2118 children) among children younger than 2 years admitted to paediatric intensive care units with extended severe acute respiratory illness (eSARI) across ten low-income and lower-middle-income countries (LMICs) that were eligible for support from Gavi, the Vaccine Alliance. Among 30 (5%) of 608 children who died from RSV infection, only 16 (53%) were provided mechanical ventilation, probably because such resources were not available at the referral hospital. These data add to findings from the Pneumonia Etiology Research for Child Health study that RSV was the most common cause of severe and very severe pneumonia in children younger than 5 years across seven LMICs^2^ and from the Child Health and Mortality Prevention Surveillance study that RSV was an important cause of death among young infants in LMICs.^3^ However, restricting enrolment to the local respiratory virus season, absence of testing for respiratory virus co-infections, and having 364 (60%) of 608 children from a single country—Nepal—might reduce the generalisability of their findings. Despite these limitations, the RSV GOLD—ICU Network study adds to increasing literature indicating that RSV is among the most common causes of severe respiratory disease in infants across all countries and income strata,^4^ and is consistent with findings from the Global Burden of Disease Study that one in every 50 deaths in children aged 0–60 months is attributable to RSV.^5^

The expanded eSARI surveillance approach described in this study complements broader RSV-surveillance initiatives, including integration of RSV sentinel surveillance into existing respiratory-virus sentinel surveillance.^6^ Routine RSV surveillance will provide crucial data to show RSV seasonality and disease burden and measure the effects of introducing RSV prevention products. In 2022 and 2023, a maternal RSV vaccine (bivalent RSV prefusion F vaccine) and a long-acting monoclonal antibody (nirsevimab) were approved by the European Medicines Agency, the US Food and Drug Administration, and other regulatory authorities on the basis of data indicating safety and efficacy in preventing severe RSV in young infants.^7,8^ Although country-specific use of products and schedules vary, early introduction of nirsevimab has shown high product effectiveness^9^ and public health effects on RSV-related and all-cause hospitalisations.^10^ Fewer countries have introduced the maternal RSV vaccine, and there are currently no published estimates of its effectiveness.

Multiple programmatic considerations remain for the introduction of RSV-prevention products, particularly in LMICs. For example, implementation of a maternal RSV vaccine would need to be aligned with antenatal-care visits. The RSV GOLD—ICU Network study study highlighted high uptake of maternal tetanus vaccination (552 [91%] of 608 mothers),^1^ which suggests integration of a maternal RSV vaccine could be feasible in Gavi-eligible countries. Tetanus vaccine can be offered at any time during pregnancy; however, clinical trials of maternal RSV vaccine targeted women during 24 to 36 weeks gestation. Due to concerns about an imbalance in preterm births in clinical trials, some regulatory agencies have recommended administration later in pregnancy until additional post-licensure safety data are available. Restriction of the time period for maternal RSV vaccination might reduce opportunities for vaccination in antenatal care and could necessitate that monoclonal-antibody products are available at birth to protect preterm infants from severe RSV disease. Perceptions of safety and real-world data on the effectiveness of maternal RSV vaccines will be important for acceptability and cost-effectiveness analyses to inform policy decisions.

Additional considerations include product pricing and availability. The Bill & Melinda Gates Foundation has supported development of an affordable, multidose, vial format of the maternal RSV vaccine, creating a method for introduction in LMICs. Long-acting monoclonal-antibody products, such as nirsevimab, are likely to remain substantially more expensive but are the only option for protecting infants at increased risk of severe RSV born to mothers who are unvaccinated. The COVID-19 vaccine rollout emphasised severe inequities in vaccine availability during 2021–22 between LMICs and high-income countries.^11^ As 97% of RSV-associated deaths in children younger than 5 years occur in LMICs,^5^ the global public health community, including governments, donors, and non-governmental organisations, should ensure that approval and introduction of RSV prevention products are prioritised. International initiatives to support affordable and equitable access to monoclonal antibodies and maternal RSV vaccines are urgently needed to protect infants in LMICs.
